# Reply to letter: Suicide in Parkinson's disease patients treated with levodopa‐carbidopa Intestinal Gel

**DOI:** 10.1002/mds.26316

**Published:** 2015-07-16

**Authors:** Hubert H. Fernandez, David G. Standaert, Krai Chatamra, Janet A. Benesh

**Affiliations:** ^1^ Center for Neurological RestorationCleveland ClinicClevelandOhioUSA; ^2^ University of Alabama at BirminghamBirminghamAlabamaUSA; ^3^ AbbVie IncNorth ChicagoIllinoisUSA

We thank Dr. Zorko and colleagues for their interest in our article and for bringing greater attention to this issue. We agree that susceptibility to suicide and suicidal ideation is a very serious issue in patients with Parkinson's disease (PD).

Our 12‐month clinical study monitored safety in 354 enrolled patients at 86 centers in 16 countries, 272 of whom completed the study. Whereas the 12‐month outcomes of this study described in our article included 2 cases of suicide (2 of 354; 0.6%), it is important to note that long‐term follow‐up of this cohort and all others in the LCIG U.S. registration program continues (n = 416, with >950 total patient‐years of follow‐up). To date, no additional cases of suicide have been reported. Both of the cases of suicide described in our report were considered “unrelated to the treatment” based on the opinion of the local study investigators responsible for the care of these patients. In agreement with the risk factors noted in the cases described by Zorko et al., both patients in the study had relevant medical histories of depression.

Although historically suicide rates were thought to be low in the PD population, more‐recent studies report a broad range of rates of suicidal ideation (0.9%–11%) and completed suicides (0.8% up to 7%).^1^, ^2^, ^3^, ^4^ None of these studies, however, address the important question of whether this suicidality relates to the disease itself or a specific medical treatment. As a cautionary example, there were a few alarming case reports and observational studies suggesting that DBS led to a higher incidence of suicide in PD patients; however, these were refuted by a larger, prospective, randomized, controlled trial showing no association between DBS and suicidal ideation or behaviors.^2^

There is much that is still unknown about the relationship between suicide, age, medical treatment, and disease in the PD patient population. Depression is a common comorbidity in PD patients, and in a multivariate analysis, severity of depression was the only predictor of suicide or death ideation for PD patients.^1^ Furthermore, depression has been associated with levodopa use and is described as an adverse event (with or without development of suicidal tendencies) in the U.S. Food and Drug Administration label for oral carbidopa/l‐dopa.^5^ Until more scientifically robust data are available, we believe that the best approach is continued vigilance in all patients for signs of depression and suicidality. At this time, the evidence for any causal relationship between suicide, PD, and treatments for PD is inconclusive.


Hubert H. Fernandez, MD,^1^* David G. Standaert, MD, PhD,^2^ Krai Chatamra, PhD,^3^ and Janet A. Benesh, BSMT^3^
^1^Center for Neurological Restoration, Cleveland Clinic, Cleveland, Ohio, USA
^2^University of Alabama at Birmingham, Birmingham, Alabama, USA
^3^AbbVie Inc., North Chicago, Illinois, USA

## Author roles

Manuscript: A. Writing of the First Draft, B. Review and Critique.

Hubert F. Fernandez: B

David G. Standaert: A, B

Krai Chatamra: B

Janet A. Benesh: A, B
